# Risk of loss to follow-up among tuberculosis patients in South Korea: whom should we focus on?

**DOI:** 10.3389/fpubh.2023.1247772

**Published:** 2023-10-19

**Authors:** Hyung Woo Kim, Jinsoo Min, Yousang Ko, Jee Youn Oh, Yun-Jeong Jeong, Eun Hye Lee, Bumhee Yang, Hyeon-Kyoung Koo, Sung-Soon Lee, Jae Seuk Park, Kwang Joo Park, Jung Hyun Chang, Joonsung Joh, Min Ki Lee, Ju Sang Kim

**Affiliations:** ^1^Division of Pulmonary and Critical Care Medicine, Department of Internal Medicine, Incheon St. Mary's Hospital, College of Medicine, The Catholic University of Korea, Seoul, Republic of Korea; ^2^Division of Pulmonary and Critical Care Medicine, Department of Internal Medicine, Seoul St. Mary’s Hospital, College of Medicine, The Catholic University of Korea, Seoul, Republic of Korea; ^3^Division of Pulmonary, Allergy and Critical Care Medicine, Department of Internal Medicine, Kangdong Sacred Heart Hospital, Hallym University College of Medicine, Seoul, Republic of Korea; ^4^Division of Pulmonary, Allergy, and Critical Care Medicine, Department of Internal Medicine, Korea University Guro Hospital, Korea University College of Medicine, Seoul, Republic of Korea; ^5^Division of Pulmonary and Critical Care Medicine, Department of Internal Medicine, Dongguk University Ilsan Hospital, Goyang, Republic of Korea; ^6^Division of Pulmonology, Allergy and Critical Care Medicine, Department of Internal Medicine, Yongin Severance Hospital, Yonsei University College of Medicine, Seoul, Republic of Korea; ^7^Division of Pulmonary and Critical Care Medicine, Department of Internal Medicine, Chungbuk National University Hospital, Chungbuk National University College of Medicine, Cheongju, Republic of Korea; ^8^Division of Pulmonary and Critical Care Medicine, Department of Internal Medicine, Ilsan Paik Hospital, Inje University College of Medicine, Goyang, Republic of Korea; ^9^Division of Pulmonary Medicine, Department of Internal Medicine, Dankook University College of Medicine, Cheonan, Republic of Korea; ^10^Department of Pulmonary and Critical Care Medicine, Ajou University School of Medicine, Suwon, Republic of Korea; ^11^Division of Pulmonary and Critical Care Medicine, Department of Internal Medicine, Mokdong Hospital, College of Medicine, Ewha Womans University, Seoul, Republic of Korea; ^12^Division of Pulmonary and Critical Care Medicine, Department of Internal Medicine, National Medical Center, Seoul, Republic of Korea; ^13^Department of Internal Medicine, Pusan National University School of Medicine, Busan, Republic of Korea

**Keywords:** tuberculosis, loss to follow-up, public-private mix, risk factors, vulnerability

## Abstract

**Introduction:**

In South Korea, public-private mix (PPM) has been a key strategy in national tuberculosis (TB) control program. This study aimed to identify rate of loss to follow-up (LTFU) among TB patients in nationwide PPM institutions and their risk factors.

**Methods:**

A nationwide prospective observational study including drug susceptible TB patients diagnosed from the 1st day to the 10th day of every month between July 2018 and December 2020 in PPM institutions was designed. Multivariable survival models in which death and failure were designated as events with competing risk were used to investigate risk factors for LTFU.

**Results:**

A total of 14,942 patients were included. Of them, 356 (2.4%) had an LTFU. Risk factors for LTFU were: underweight patients (adjusted hazard ratio (aHR): 1.47, 95% CI: 1.12–1.92), patients living alone (aHR: 1.43, 95% CI: 1.16–1.76), heavy drinkers (aHR: 1.67, 95% CI: 1.16–2.39), those with malignancy (aHR: 1.49, 95% CI: 1.07–2.05), foreigners (aHR: 5.96, 95% CI: 4.51–7.89), and those with previous TB history reported as an unfavorable outcome (aHR: 4.43, 95% CI: 2.77–7.08). Effect of age on LTFU was not significant. Brief interruption of anti-TB treatment (less than two months) in current session was associated with subsequent LTFU [adjusted odds ratio: 13.09 (10.29–16.66)].

**Conclusion:**

Identifying vulnerability of patients such as living alone, being heavy alcoholics, being foreigners or having previous TB history reported as an unfavorable outcome is required. Thorough case management for these vulnerable groups could be feasible with collaboration between public and private sectors.

## 1. Introduction

Tuberculosis is still a global threat. Approximately 1.6 million deaths were attributed to TB in 2021 globally (1). To reduce TB burden, a target of more than 90% of treatment success rate until 2025 has been suggested (2), although it remained at 86% globally in 2021 (1). For a successful treatment, maintaining adherence is essential. Poor adherence can lead to prolonged infectiousness, acquired drug resistance, relapse, and even death (3). Therefore, keeping patients’ adherence to anti-TB treatment is underscored in national TB control programs (NTP).

In South Korea, public-private mix (PPM) is a key strategy of NTP as most patients with TB in South Korea are managed in private sectors (4). PPM project was introduced in 2011 to resolve the stagnation in decline of TB incidence in early 2000’s due to a low treatment success rate in private hospitals (5). TB specialist nurses were dispatched to 184 PPM hospitals in 21 districts located all over the country to manage patients’ adherence. After implementation of the PPM project, TB incidence in South Korea has gradually decreased – from 100.8 cases per 100,000 population in 2011 to 44.6 cases per 100,000 population in 2021 (6).

In our previous study, we have reported reasons for loss to follow-up (LTFU) in TB patients in South Korea (7). Additionally, we have investigated risk factors for LTFU at the early stage of the PPM project (2011–2014) (8). However, these studies were basically cross-sectional or retrospective. In this nationwide prospective cohort study, we collected data in a time-to event form and analyzed risk factors for LTFU to identify which group we should focus on to prevent LTFU.

## 2. Materials and methods

### 2.1. Data source and study population

To improve management of patients in PPM institutions, TB specialist nurses in each PPM institution report several indicators for TB management and treatment outcome of enrolled TB patients to Korean Disease Control and Prevention Agency (KDCA) (9). In addition, to identify clinical characteristics of TB patients managed in PPM institutions, Korean TB cohort (KTBC), a prospective observational cohort, has been implemented since September 2018 (10, 11). Every TB patient notified from the first day to the tenth day in a month was automatically enrolled in KTBC on the date of TB notification. TB specialist nurses of 172 hospitals located all over the country collected baseline information with a prespecified case reporting form. Additional data such as results of 2-month sputum examination and those of drug susceptibility test (DST) were collected during anti-TB treatment. Final treatment outcome of each patient was reported at one year after the date of enrollment. Regional and central data managers check missing, incorrect, or inappropriate data every quarter of a year to improve data quality (10, 11).

In this study, TB patients enrolled in KTBC from September 2018 to December 2020 were included. Exclusion criteria were: (1) patients with reported rifampicin resistance from either genotype or phenotype DST, (2) patients who were enrolled after transfer from other hospitals, (3) patients who initiated anti-TB treatment at other hospitals, (4) patients who were finally diagnosed as not TB and reported as ‘diagnosis changed’.

### 2.2. Exposure variables

In KTBC, baseline demographic features such as age, sex, weight, height, families living together, nationality, smoking status, and alcohol habit were collected. Patients were classified into five age groups (< 20 years, 20–34 years, 35–49 years, 50–64 years, and ≥ 65 years). Body mass index (BMI) was calculated and categorized into three groups: underweight (BMI < 18.5 kg/m^2^), normal or overweight (BMI = 18.5–25 kg/m^2^), and obesity (BMI ≥ 25 kg/m^2^) (12). Heavy drinking was defined as more than eight drinks per week for males and more than four drinks per week for females (13). Underlying medical comorbidities such as diabetes mellitus (DM), chronic pulmonary, cardiovascular, hepatic, renal disease, neurologic disease, and autoimmune disease were surveyed. Initial TB-related symptoms such as cough, sputum, dyspnea, chest pain, hemoptysis, fever, general weakness, and weight loss were reported in KTBC. Six categories by previous TB treatment history were used: new patients, relapse, previous treatment failure, previous LTFU, other previously treated, and those with uncertain previous TB history (14). A bacteriologically confirmed TB case was defined as one who showed at least one positive result in smear microscopy, culture, or WHO-approved rapid diagnostic. Pulmonary TB (PTB) was defined as TB involving lung parenchyma or tracheobronchial tree. TB involving other organs was classified as extrapulmonary TB (EPTB). Patients with both PTB and EPTB were classified as PTB. A regimen of rifampin, isoniazid, ethambutol, and pyrazinamide was defined as standard regimen. Other compositions of first-line or second-line anti-TB medication were classified as modified regimen. Isoniazid mono-resistant TB (HrTB) was diagnosed based on results of either genotype drug susceptibility test (DST) or phenotype DST.

Patients who were still on treatment at one year after treatment initiation were classified as still on treatment regardless of a reason for prolonged treatment. For the rest of the cohort, one of the five treatment outcomes (treatment success, failure, LTFU, death, and transfer out) was assigned, which was defined according to the WHO’s criteria (14). Beside LTFU defined as treatment interruption for two consecutive months or more, any consecutive treatment interruption for a week or more but less than 2 months was investigated retrospectively at the time of outcome report for patients whose treatment outcome was reported as ‘treatment success’ or ‘LTFU’.

### 2.3. Statistical analysis

Patients enrolled in KTBC were prospectively followed up from the date of enrollment to the last date of anti-TB treatment in institutions where patients were enrolled. The rate of LTFU was calculated by each baseline demographic and clinical feature. Risk factors for LTFU were investigated in a time-to-event model considering competing risk using the Fine and Gray method (15). In our model, ‘LTFU’ was the outcome of interest, whereas ‘death’ and ‘failure’ were outcomes with competing risk. Other outcomes such as ‘treatment success’, ‘transfer out’, and ‘still on treatment’ were censored. To estimate the effect of prior treatment interruption for less than two months, a case (LTFU group) – control (treatment success group) study within a cohort was designed. Univariable and multivariable logistic regression analyses were performed, and odds ratio was presented. All statistical analyses were conducted with RStudio version 1.2.5033. Statistical significance was considered when two-sided *p*-value was less than 0.05.

### 2.4. Ethics statement

The Institutional Review Board (IRB) of Incheon St. Mary’s Hospital, the Catholic University of Korea approved the study protocol (IRB No. OC21ZNSI0063). KDCA has the authority to collect and analyze data for public health and research purposes according to the Tuberculosis Prevention Act. All patients’ records were previously anonymized. The need for Informed consent was waived by the IRB because this study was observational, and no patient was at risk for personal information leakage.

## 3. Results

**Figure 1:**
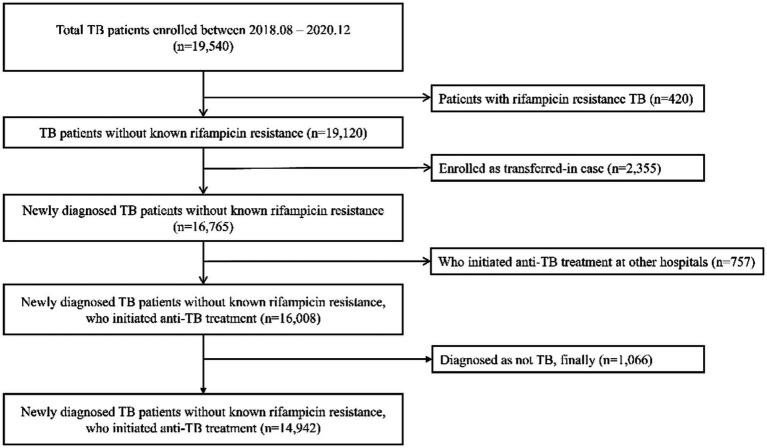
Flow chart showing patient enrollment.

Among TB patients enrolled during the study period, a total of 14,942 patients were finally included (Figure 1). Of them, 356 patients had LTFU. Baseline demographic and clinical features of included patients by six treatment outcomes are presented in Table 1. Mean age of the LTFU group was 56.3 ± 18.9 years, which was lower than that of total patients (61.0 ± 19.0 years). In the LTFU group, proportion of patients who were living alone (50.6%), foreigners (21.3%), and heavy drinkers (10.7%) were higher than those in total patients. Among medical comorbidities, chronic liver disease (3.4%) and malignancy (12.1%) had higher proportions than others in total patients. Asymptomatic patients accounted for 32.3% of the LTFU group, which was not quite different from that in total patients (31.3%). As for past TB history, previous LTFU and unknown TB history accounted for 6.5 and 3.4% in the LTFU group, respectively, which were higher than those in total patients (1.2 and 1.1%, respectively). In the LTFU group, EPTB accounted for 36.3%, higher than that in total patients (23.5%). Proportion of bacteriologically confirmed TB was 64.5% in the LTFU group, which was lower than that (77.2%) in total patients.

**Table 1:** Baseline demographic and clinical features of enrolled TB patients by treatment outcome.

	Treatment success	Failure	LTFU	Death	Transfer-out	Still on treatment	Total
Total	10,368 (100.0)	10 (100.0)	356 (100.0)	1,469 (100.0)	1769 (100.0)	970 (100.0)	14,942 (100.0)
Age (mean ± S.D.)	58.9 ± 18.8	61.6 ± 18.4	56.3 ± 18.9	74.4 ± 14.3	65.1 ± 18.9	58.0 ± 17.6	61.0 ± 19.0
Sex							
Male	6,178 (59.6)	7 (70.0)	216 (60.7)	932 (63.4)	1,054 (59.6)	589 (60.7)	8,976 (60.1)
Female	4,190 (40.4)	3 (30.0)	140 (39.3)	537 (36.6)	715 (40.4)	381 (39.3)	5,966 (39.9)
BMI (kg/m^2^, mean ± S.D.)	22.0 ± 3.3	20.8 ± 3.9	21.5 ± 3.6	20.4 ± 3.8	21.3 ± 3.5	21.7 ± 3.5	21.7 ± 3.5
Family							
Living with family	6,730 (64.9)	7 (70.0)	176 (49.4)	798 (54.3)	898 (50.8)	581 (59.9)	9,190 (61.5)
Living alone	3,638 (35.1)	3 (30.0)	180 (50.6)	671 (45.7)	871 (49.2)	389 (40.1)	5,752 (38.5)
Nationality							
Korean	9,989 (96.3)	10 (100.0)	280 (78.7)	1,442 (98.2)	1710 (96.7)	929 (95.8)	14,360 (96.1)
Foreigner	379 (3.7)	0 (0)	76 (21.3)	27 (1.8)	59 (3.3)	41 (4.2)	582 (3.9)
Alcohol habit							
No drinking	6,571 (63.4)	7 (70.0)	233 (65.4)	1,119 (76.2)	1,230 (69.5)	629 (64.8)	9,789 (65.5)
Social drinking	3,201 (30.9)	2 (20.0)	85 (23.9)	238 (16.2)	403 (22.8)	268 (27.6)	4,197 (28.1)
Heavy drinking	596 (5.7)	1 (10.0)	38 (10.7)	112 (7.6)	136 (7.7)	73 (7.5)	956 (6.4)
Smoking							
Non-smoker	6,150 (59.3)	5 (50.0)	214 (60.1)	960 (65.4)	1,105 (62.5)	562 (57.9)	8,996 (60.2)
Ex-smoker	2065 (19.9)	3 (30.0)	60 (16.9)	325 (22.1)	332 (18.8)	161 (16.6)	2,946 (19.7)
Current smoker	2,153 (20.8)	2 (20.0)	82 (23.0)	184 (12.5)	332 (18.8)	247 (25.5)	3,000 (20.1)
Comorbidity							
DM	1913 (18.5)	2 (20.0)	65 (18.3)	428 (29.1)	441 (24.9)	194 (20.0)	3,043 (20.4)
Chronic pulmonary disease	497 (4.8)	1 (10.0)	9 (2.5)	119 (8.1)	101 (5.7)	54 (5.6)	781 (5.2)
Cardiovascular disease	502 (4.8)	1 (10.0)	7 (2.0)	171 (11.6)	127 (7.2)	45 (4.6)	853 (5.7)
Chronic liver disease	192 (1.9)	0 (0)	12 (3.4)	51 (3.5)	44 (2.5)	35 (3.6)	334 (2.2)
Chronic renal disease	266 (2.6)	0 (0)	12 (3.4)	156 (10.6)	85 (4.8)	30 (3.1)	549 (3.7)
Chronic neurologic disease	756 (7.3)	0 (0)	20 (5.6)	331 (22.5)	266 (15.0)	64 (6.6)	1,437 (9.6)
Malignancy	850 (8.2)	1 (10.0)	43 (12.1)	286 (19.5)	137 (7.7)	73 (7.5)	1,390 (9.3)
Autoimmune disease	108 (1.0)	1 (10.0)	5 (1.4)	19 (1.3)	21 (1.2)	20 (2.1)	174 (1.2)
TB-related symptom							
Asymptomatic	3,494 (33.7)	3 (30.0)	115 (32.3)	335 (22.8)	480 (27.1)	247 (25.5)	4,674 (31.3)
With TB-related symptom	6,874 (66.3)	7 (70.0)	241 (67.7)	1,134 (77.2)	1,289 (72.9)	723 (74.5)	10,268 (68.7)
Cough/sputum	3,811 (36.8)	5 (50.0)	107 (30.1)	461 (31.4)	658 (37.2)	359 (37.0)	5,401 (36.1)
Dyspnea	1,619 (15.6)	3 (30.0)	46 (12.9)	483 (32.9)	349 (19.7)	114 (11.8)	2,614 (17.5)
Chest pain	875 (8.4)	0 (0)	20 (5.6)	74 (5.0)	124 (7.0)	57 (5.9)	1,150 (7.7)
Hemoptysis	440 (4.2)	1 (10.0)	15 (4.2)	40 (2.7)	66 (3.7)	38 (3.9)	600 (4.0)
Fever	1,220 (11.8)	1 (10.0)	41 (11.5)	264 (18.0)	316 (17.9)	134 (13.8)	1976 (13.2)
General weakness	317 (3.1)	0 (0)	20 (5.6)	207 (14.1)	154 (8.7)	46 (4.7)	744 (5.0)
Weight loss	732 (7.1)	1 (10.0)	21 (5.9)	102 (6.9)	149 (8.4)	91 (9.4)	1,096 (7.3)
Past TB history							
New patients	8,877 (85.6)	6 (60.0)	277 (77.8)	1,221 (83.1)	1,475 (83.4)	744 (76.7)	12,600 (84.3)
Relapse	1,280 (12.3)	3 (30.0)	44 (12.4)	202 (13.8)	235 (13.3)	189 (19.5)	1953 (13.1)
Previous LTFU	85 (0.8)	1 (10.0)	23 (6.5)	20 (1.4)	29 (1.6)	18 (1.9)	176 (1.2)
Previous failure	9 (0.1)	0 (0)	0 (0)	2 (0.1)	2 (0.1)	6 (0.6)	19 (0.1)
Other previously treated	22 (0.2)	0 (0)	0 (0)	3 (0.2)	7 (0.4)	2 (0.2)	34 (0.2)
Unknown TB history	95 (0.9)	0 (0)	12 (3.4)	21 (1.4)	21 (1.2)	11 (1.1)	160 (1.1)
TB site							
PTB	7,153 (69.0)	9 (90.0)	204 (57.3)	1,076 (73.2)	1,280 (72.4)	600 (61.9)	10,322 (69.1)
EPTB	2,490 (24.0)	0 (0)	128 (36.0)	269 (18.3)	352 (19.9)	273 (28.1)	3,512 (23.5)
PTB + EPTB	725 (7.0)	1 (10.0)	24 (6.7)	124 (8.4)	137 (7.7)	97 (10.0)	1,108 (7.4)
Initial treatment regimen							
HREZ	9,819 (94.7)	10 (100.0)	316 (88.8)	1,231 (83.8)	1,603 (90.6)	848 (87.4)	13,827 (92.5)
HRE	326 (3.1)	0 (0)	22 (6.2)	130 (8.8)	100 (5.7)	43 (4.4)	621 (4.2)
Other modified regimen	223 (2.2)	0 (0)	18 (5.1)	108 (7.4)	66 (3.7)	79 (8.1)	494 (3.3)
Resistance pattern							
HrTB	422 (4.1)	0 (0)	15 (4.2)	48 (3.3)	87 (4.9)	106 (10.9)	678 (4.5)
Results of microbiological or radiological exam among patients with PTB							
Smear-positive	2,269 (28.8)	5 (50.0)	62 (27.2)	508 (42.3)	498 (35.1)	288 (41.3)	3,630 (31.8)
Culture-positive	5,268 (66.9)	8 (80.0)	123 (53.9)	844 (70.3)	996 (70.3)	523 (75.0)	7,762 (67.9)
Bacteriologically-confirmed	5,945 (75.5)	8 (80.0)	147 (64.5)	998 (83.2)	1,142 (80.6)	583 (83.6)	8,823 (77.2)
Cavitary TB	1731 (22.0)	4 (40.0)	68 (29.8)	251 (20.9)	332 (23.4)	248 (35.6)	2,634 (23.0)

TB, tuberculosis; LTFU, loss to follow-up; S.D., standard deviation; BMI, body mass index; DM, diabetes mellitus; PTB, pulmonary tuberculosis; EPTB, extrapulmonary tuberculosis; Hr-TB, isoniazid mono-resistant tuberculosis.

### 3.1. LTFU rate by baseline demographic and clinical features

**Figure 2:**
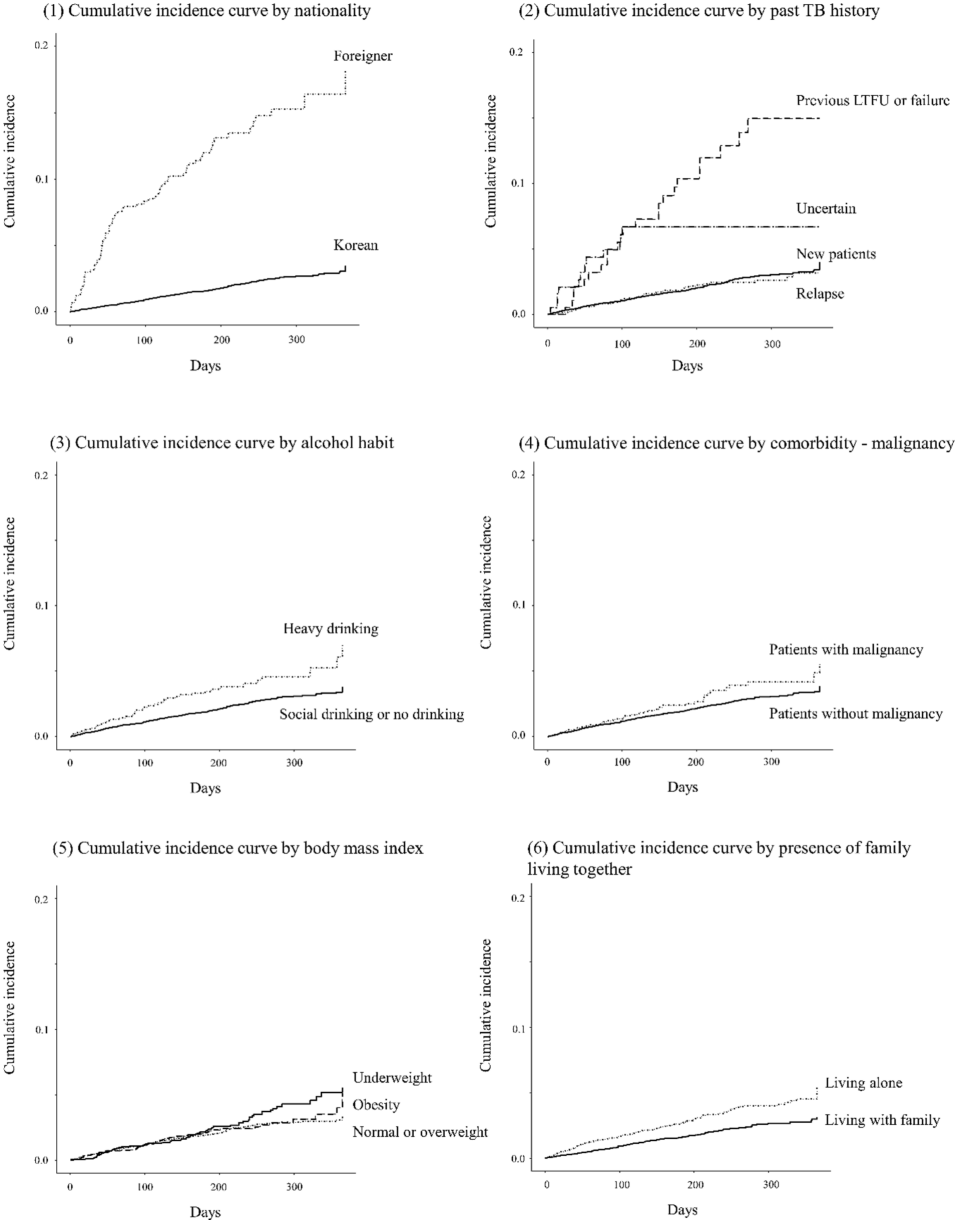
Cumulative incidence curves for loss to follow-up by several baseline features.

Patients aged 20–34 years showed the highest LTFU rate (68.3 per 1,000 person-years) followed by those aged 50–64 years (50.7 per 1,000 person-years; Table 2). LTFU rate in older adult patients was lower (33.8 per 1,000 person-years) than in other age groups. TB patients who were underweight (59.6 per 1,000 person-years), those living alone (60.3 per 1,000 person-years), heavy drinkers (73.5 per 1,000 person-years), and foreigners (247.2 per 1,000 person-years) showed high LTFU rates. Additionally, patients with chronic liver disease (65.3 per 1,000 person-years), those with malignancy (62.6 per 100 person-years), those who complained of general weakness (62.4 per 1,000 person-years), those with previous TB history reported as unfavorable outcome (LTFU or failure) (212.8 per 1,000 person-years), and those with uncertain TB history (129.4 per 1,000 person-years) showed high LTFU rates. LTFU rates in patients with clinically diagnosed TB (58.9 per 1,000 person-years), those with EPTB (64.9 per 1,000 person-years), and those treated with modified regimen (67.7 per 100 person-years) were also relatively high. Cumulative incidence curves for LTFU by several baseline features are presented in Figure 2.

**Table 2:** Rate of loss to follow-up among enrolled TB patients by baseline demographic and clinical variables.

Variable	LTFU (*n*)	Total (*n*)	Total follow-up (years)	LTFU rate (per 1,000 person-years)
Age				
<20	4	203	116.4	34.4
20-34	59	1,499	863.8	68.3
35-49	60	2,296	1354.8	44.3
50-64	116	3,995	2289.2	50.7
≥65	117	6,949	3458.6	33.8
Sex				
Male	216	8,976	4860.3	44.4
Female	140	5,966	3222.6	43.4
BMI				
Underweight (<18.5 kg/m^2^)	73	2,472	1224.5	59.6
Normal or overweight (18.5–25 kg/m^2^)	227	10,213	5596.9	40.6
Obesity (≥25 kg/m^2^)	56	2,257	1261.5	44.4
Family				
Living with family	176	9,190	5096.0	34.5
Living alone	180	5,752	2986.8	60.3
Alcohol habit				
No drinking or social drinking	318	13,986	7565.6	42.0
Heavy drinking	38	956	517.2	73.5
Smoking				
Non-smoker	214	8,996	4772.8	44.8
Ex-smoker	60	2,946	1578.1	38.0
Current smoker	82	3,000	1731.9	47.3
Comorbidities				
DM	65	3,043	1583.5	41.0
chronic pulmonary disease	9	781	405.6	22.2
Cardiovascular disease	7	853	414.5	16.9
Chronic liver disease	12	334	183.9	65.3
Chronic renal disease	12	549	249.5	48.1
Chronic neurologic disease	20	1,437	635.3	31.5
Malignancy	43	1,390	687.1	62.6
Autoimmune disease	5	174	100.3	49.8
Nationality				
Korean	280	14,360	7775.4	36.0
Foreign	76	582	307.5	247.2
Symptoms				
Asymptomatic patients	115	4,674	2570.1	44.7
Patients with TB-related symptom	241	10,268	5512.7	43.7
Cough/sputum	107	5,401	2958.3	36.2
Dyspnea	46	2,614	1268.4	36.3
Chest pain	20	1,150	629.7	31.8
Hemoptysis	15	600	341.5	43.9
Fever	41	1976	1001.6	40.9
General weakness	20	744	320.4	62.4
Weight loss	21	1,096	614.6	34.2
Past TB history				
New patients	277	12,600	6744.8	41.1
Relapse	44	1953	1137.2	38.7
Previous unfavorable outcome (LTFU or failure)	23	195	108.1	212.8
Uncertain^a^	12	194	92.7	129.4
Results of microbiological examinations				
Smear (+), bacteriologically confirmed PTB	62	3,630	1960.8	31.6
Smear (−), bacteriologically confirmed PTB	92	5,422	2894.1	31.8
Clinically diagnosed PTB	74	2,378	1255.7	58.9
EPTB	128	3,512	1972.3	64.9
Resistance pattern and regimen type				
DS-TB, standard regimen	302	13,194	7071.8	42.7
DS-TB, modified regimen	39	1,070	576.2	67.7
Hr-TB	15	678	434.7	34.5

^a^Patients with unknown TB history or patients with past TB history whose treatment outcome was uncertain. LTFU, loss to follow-up; TB, tuberculosis; HR, hazard ratio; CI, confidence interval; BMI, body mass index; DM, diabetes mellitus; PTB, pulmonary tuberculosis; EPTB, extrapulmonary tuberculosis; DS-TB; drug-susceptible tuberculosis, Hr-TB, isoniazid mono-resistant tuberculosis.

### 3.2. Risk factor for LTFU

Results of a multivariable survival analysis using explanatory variables such as age, sex, and significant (*p* < 0.100) variables in univariable analysis are presented in Table 3. Effect of age on LTFU was insignificant. Risk factors for LTFU were: patients who were underweight (adjusted hazard ratio (aHR): 1.47, 95% CI: 1.12–1.92, *p* = 0.005), those living alone (aHR: 1.43, 95% CI: 1.16–1.76, *p* = 0.001), heavy drinkers (aHR: 1.67, 95% CI: 1.16–2.39, *p* = 0.005), those with malignancy (aHR: 1.49, 95% CI: 1.07–2.05, *p* = 0.017), foreigners (aHR: 5.96, 95% CI: 4.51–7.89, *p* < 0.001), those with previous TB history reported as unfavorable outcome (aHR: 4.43, 95% CI: 2.77–7.08, *p* < 0.001), those with uncertain TB history (aHR: 2.81, 95% CI: 1.52–5.19, *p* = 0.001), those with clinically diagnosed PTB (aHR: 1.59, 95% CI: 1.16–2.18, *p* = 0.004), and those with EPTB (aHR: 2.01, 95%CI: 1.51–2.670, *p* < 0.001).

**Table 3:** Risk factors for LTFU among enrolled TB patients.

Variable	Univariable analysis		Multivariable analysis	
	HR (95% CI)	*p* value	HR (95% CI)	*p* value
Age				
<20	1		1	
20-34	1.97 (0.72–5.41)	0.187	1.20 (0.43–3.31)	0.729
35-49	1.27 (0.46–3.48)	0.643	1.06 (0.38–2.92)	0.917
50-64	1.42 (0.53–3.85)	0.485	1.36 (0.50–3.72)	0.553
≥65	0.86 (0.32–2.32)	0.766	1.07 (0.39–2.94)	0.903
Sex				
Male	1		1	
Female	0.99 (0.80–1.22)	0.902	1.02 (0.82–1.27)	0.843
BMI				
Underweight (<18.5 kg/m^2^)	1.33 (1.03–1.74)	0.031	1.47 (1.12–1.92)	0.005
Normal or overweight (18.5–25.0 kg/m^2^)	1		1	
Obesity (≥25.0 kg/m^2^)	1.12 (0.83–1.50)	0.461	1.09 (0.81–1.47)	0.564
Family				
Living with family	1		1	
Living alone	1.69 (1.37–2.08)	<0.001	1.43 (1.16–1.76)	0.001
Alcohol habit				
No drinking or social drinking	1		1	
Heavy drinking	1.73 (1.23–2.42)	0.001	1.67 (1.16–2.39)	0.005
Smoking				
Non-smoker	1			
Ex-smoker	0.85 (0.64–1.13)	0.262		
Current smoker	1.10 (0.86–1.42)	0.444		
Comorbidities				
DM	0.88 (0.67–1.15)	0.339		
Chronic pulmonary disease	0.47 (0.24–0.90)	0.023	0.62 (0.32–1.21)	0.162
Cardiovascular disease	0.33 (0.16–0.71)	0.004	0.47 (0.22–0.99)	0.047
Chronic liver disease	1.45 (0.82–2.57)	0.206		
Chronic renal disease	0.92 (0.52–1.64)	0.776		
Chronic neurologic disease	0.60 (0.38–0.94)	0.026	0.76 (0.48–1.21)	0.250
Malignancy	1.34 (0.97–1.84)	0.072	1.49 (1.07–2.05)	0.017
Autoimmune disease	0.88 (0.67–1.15)	0.339		
Nationality				
Korean	1		1	
Foreign	7.11 (5.50–9.18)	<0.001	5.96 (4.51–7.89)	<0.001
Symptoms				
Any TB-related symptom (*vs* asymptomatic)	0.94 (0.75–1.18)	0.592		
Cough/sputum	0.76 (0.60–0.95)	0.016	1.06 (0.83–1.35)	0.666
Dyspnea	0.72 (0.53–0.98)	0.037	0.81 (0.58–1.11)	0.191
Chest pain	0.73 (0.46–1.14)	0.17		
Hemoptysis	1.03 (0.61–1.72)	0.921		
Fever	0.88 (0.64–1.22)	0.437		
General weakness	1.17 (0.75–1.83)	0.487		
Weight loss	0.77 (0.49–1.19)	0.241		
Past TB history				
New patients	1		1	
Relapse	0.95 (0.69–1.31)	0.756	0.98 (0.71–1.36)	0.914
Previous unfavorable outcome (LTFU or failure)	5.15 (3.36–7.89)	<0.001	4.43 (2.77–7.08)	<0.001
Uncertain^a^	3.01 (1.67–5.43)	<0.001	2.81 (1.52–5.19)	0.001
Results of microbiological examinations				
Smear (+), bacteriologically confirmed PTB	0.96 (0.70–1.33)	0.809	0.87 (0.63–1.20)	0.400
Smear (−), bacteriologically confirmed PTB	1		1	
Clinically diagnosed PTB	1.90 (1.40–2.59)	<0.001	1.59 (1.16–2.18)	0.004
EPTB	2.09 (1.60–2.73)	<0.001	2.01 (1.51–2.67)	<0.001
Resistance pattern and regimen type				
DS-TB, standard regimen	1		1	
DS-TB, modified regimen	1.45 (1.04–2.03)	0.030	1.36 (0.94–1.95)	0.100
Hr-TB	0.83 (0.50–1.40)	0.488	0.86 (0.51–1.45)	0.560

^a^Patients with unknown TB history or patients with past TB history whose treatment outcome was uncertain. LTFU, loss to follow-up; TB, tuberculosis; HR, hazard ratio; CI, confidence interval; BMI, body mass index; DM, diabetes mellitus; PTB, pulmonary tuberculosis; EPTB, extrapulmonary tuberculosis; DS-TB; drug-susceptible tuberculosis, Hr-TB, isoniazid mono-resistant tuberculosis.

In a multivariable logistic regression model of case–control study, any consecutive interruption of anti-TB treatment for a week or more but less than two months was associated with subsequent LTFU [adjusted odds ratio (aOR): 13.09, 95% CI: 10.29–16.66, *p* < 0.001]. Furthermore, the effect of self-interruption on subsequent LTFU was significant (aOR: 18.01, 95% CI: 13.57–23.90, *p* < 0.001).

## 4. Discussion

In this study, we identified several groups showing high LTFU risk, including foreigners, patients with previous TB history reported as unfavorable outcome or those with uncertain TB history, those living alone, those with malignancy, and those who were underweight and heavy drinkers. Effect of age on LTFU was insignificant. In addition, prior treatment interruption, especially self-interruption during current TB episode, was associated with subsequent LTFU.

Previous LTFU has been found to be a significant risk factor for LTFU in previous studies (8, 16, 17), suggesting that reasons for previous LTFU are not easily improved, which might continuously affect LTFU during following treatment. We also found that a short interruption of anti-TB treatment heralded a subsequent LTFU. Directly observed treatment (DOT) is not widely implemented in South Korea due to the lack of manpower (18). Although patients with non-compliance are managed with telephone consultation and home visit, prevention of non-compliance in advance is more efficient in reducing transmission and development of drug-resistance than managing non-complaint patients which already occurred (19). Various digital adherence technologies could be applied selectively to TB patients with previous LTFU or those with short treatment interruption which can herald subsequent LTFU (Table 4).

**Table 4:** Estimated effects of any consecutive treatment interruption or self-interruption for more than a week but less than two months during anti-TB treatment on subsequent loss to follow-up [presented as odds ratios from case (LTFU group) – control (treatment success group) study].

	Treatment success group (*n* = 10,368)	LTFU group (*n* = 356)	Univariable analysis		Multivariable analysis^a^	
			OR (95% CI)	*p* value	adjusted OR (95% CI)	*p* value
Any consecutive interruption of anti-TB treatment for 7–60 days	945 (9.1%)	187 (52.5%)	11.03 (8.87–13.73)	<0.001	13.09 (10.29–16.66)	<0.001
Any consecutive self-interruption of anti-TB treatment for 7–60 days	271 (2.6%)	108 (30.3%)	16.23 (12.56–20.96)	<0.001	18.01 (13.57–23.90)	<0.001

^a^Adjusted for age, sex, nationality, having a family living together, alcohol habit, body mass index, having chronic lung disease, having cardiovascular disease, having chronic liver disease, having malignancy, presenting with cough/sputum, presenting with chest pain, presenting with general weakness, past TB history, results of microbiological examinations, resistance pattern, and regimen type, which were significant in univariable analysis. TB, tuberculosis; LTFU, loss to follow-up; OR, odds ratio; CI, confidence interval.

In addition to DOT, medical or socioeconomic conditions associated with previous LTFU should be thoroughly investigated for each patient. In a previous study, adverse effects of anti-TB treatment were the most frequent causes for LTFU (7). Gastrointestinal discomfort was the most frequent adverse effect among TB patients in South Korea (20). Although there are recommendations for managing gastrointestinal discomfort in Korean guidelines for TB, such as modifying medication time after meal or before sleep, these solutions are mostly empirical. Additionally, efficacy and adequate duration of modified regimens such as rifampin-sparing regimen for patients who are not tolerable to rifampin are unknown (21). Evidence-based management of adverse effects of anti-TB treatment should be investigated and applied in clinical practice. Inadequate knowledge, attitude, and belief about TB would lead to patients’ refusal treatment (22), which is another major cause for LTFU (7). Enforced patient education and counseling program should be implemented, especially for patients with previous LTFU history and those with short treatment interruption during current TB treatment. Although rate of LTFU in each PPM hospital and each district is monitored as an key indicator for TB control in the PPM project (23), LTFU rate among patients with previous LTFU history should also be underscored.

Low BMI was associated with unfavorable outcome, especially death, in previous studies (24, 25). We found that LTFU risk was increased for TB patients who were underweight. Considering that patients with low BMI show slower gastric emptying and heightened visceral perception than those with normal or high BMI (26), the incidence of gastrointestinal discomfort during anti-TB treatment might be relatively high among TB patients who are underweight, which might have contributed to the high LTFU risk. Gastrointestinal discomfort was the most frequent adverse effect among TB patients and those who have LTFU (7, 20). Similarly, we presume that the high LTFU rate among TB patients with malignancy could be attributable to gastrointestinal discomfort which can be aggravated by anti-cancer chemotherapy. Moreover, anti-TB treatment could be easily interrupted due to minor adverse effects among TB patients with terminal cancer (7). High LTFU rate among these two groups underline the significance of managing adverse effect of anti-TB treatment and the role of medical staffs in PPM hospitals.

Foreigners showed higher LTFU rate than Korean TB patients in previous studies (8, 27). In the present study, we identified dozens of foreign TB patients who returned to their own countries without informing their attending medical staffs and reported as LTFU thereafter. As migration itself is a known risk factor for LTFU (28). Thus, thorough management during international transfer-out is needed, which requires cooperation with immigration offices. Moreover, considering that accessibility to healthcare services is often limited for immigrants due to difficulties in communication (29), effects of patient education and counseling might be insufficient for foreign patients. Burden of medical cost might also have an effect. Although all foreigners who stay in South Korea for 6 months or more are subjected to subscription to National Health Insurance (NHI) since 2019, proportion of foreigners enrolled in NHI remained at 76.8% of 1.6 million registered foreigners in 2021 (30). Since 2017, NHI has paid for full medical cost related to TB treatment (18). Considering that the role of public health centers in provision of curative services is limited in South Korea (31), medical cost could be a hurdle that limits visiting private hospitals among foreign TB patients not enrolled in NHI and undocumented foreigners with a number estimated to be 0.4 million in 2021 (32).

We identified TB patient living alone and heavy alcoholics as additional vulnerable groups for LTFU. Role of family support is crucial for patient adherence to treatment by providing emotional support, motivation and supervising patients’ medication (22, 33). However, due to rapid increase in nuclear families, the number of older adult people living alone is rising in South Korea. This leads to the lack of healthcare, poverty, social alienation, and depression among older adult population (34), which are all risk factors for non-adherence to treatment. In addition, heavy alcohol use is a well-known risk factor for LTFU (35). As in TB patients living alone, heavy alcohol use is a result of social marginalization (36), which is another major reason for LTFU (7). To tackle down these vulnerable groups, the government of South Korea has implemented enhanced case management based on vulnerability assessment since 2022 (37). In this vulnerability assessment, whether the patient has physical disability, difficulties in communication, psychological problem including substance abuse, any other comorbidities, difficulties in visiting hospitals, families living together, and adequate residence is investigated (38). For medical staff in private hospitals, these factors are difficult problems to deal with. Government’s role should be underscored in this issue. Additionally, beyond just identifying these vulnerabilities, feasible solutions should be investigated and assessed.

Interestingly, age was not associated with LTFU risk, which was discordant with results of a previous Korean study covering LTFU cases in the early period of the PPM project (2011–2014) (8). In that study, old age was associated with a high LTFU risk. Although direct comparison between the two studies is unfeasible due to different study design, decrease in LTFU between the two study periods was the most prominent among older adult TB patients. Older adult TB patients have a higher risk of adverse effects of anti-TB treatment (39). Adverse effects are known to be the most common reasons for LTFU (7). However, we presume that effects of active management of adverse effect, counseling, and patient education in PPM hospitals are the most prominent in older adult TB patient and successful implement of PPM project might in part contribute to a decrease in LTFU (40).

This study is the first prospective observational study investigating LTFU among nationwide TB patients in South Korea. However, it has several limitations. First, patients’ socioeconomic status such as income level was not investigated, although it was a significant factor for LTFU (41). Second, although a substantial proportion (11.8%) of enrolled patients were transferred out during the study period, their outcomes after transfer-out were not investigated. A previous study has shown hidden LTFU cases in South Korea during the process of inter-hospital transfer (8). However, as status of re-registration at transfer-in hospitals has been thoroughly checked by TB specialist nurses in initial hospitals since 2016, we assume that the risk of LTFU during process of transfer is insignificant as in the previous study. Third, although high-risk groups for LTFU were demonstrated in this study, the reasons and determinant factors for LTFU in detail were not covered in this study. Further studies are needed to investigate how to reduce LTFU risk among these high-risk patients by knowing reasons for LTFU.

In conclusion, previous LTFU history and brief interruption of anti-TB treatment in current treatment session are predictors for subsequent LTFU, which require enhanced case management. TB patients who were underweight and those with malignancy were high-risk groups. Thus, PPM hospitals should have an active management for adverse effects of anti-TB treatment. Foreigners, those living alone, and heavy alcoholics were vulnerable groups. For these groups, the role of government is underscored to prevent LTFU. Collaboration between private and public sectors is required.

## Data availability statement

The raw data supporting the conclusions of this article will be made available by the authors, without undue reservation.

## Ethics statement

The studies involving humans were approved by the Institutional Review Board (IRB) of Incheon St. Mary’s Hospital, the Catholic University of Korea. The studies were conducted in accordance with the local legislation and institutional requirements. The ethics committee/institutional review board waived the requirement of written informed consent for participation from the participants or the participants’ legal guardians/next of kin because this study was observational, and no patient was at risk for personal information leakage.

## Author contributions

JK, HK, S-SL, and JP designed the study. KP, JC, JJ, and ML contributed to data collection. HK, YK, JO, and H-KK cleaned and verified the dataset. HK did the statistical analysis and wrote the manuscript. Y-JJ, EL, and BY interpreted the results. JK and JM reviewed and edited the manuscript. JK supervised the work. All authors had full access to all the data in the study and had final responsibility for the decision to submit for publication.
